# Time to recovery from COVID-19 and its predictors among patients admitted to treatment center of Wollega University Referral Hospital (WURH), Western Ethiopia: Survival analysis of retrospective cohort study

**DOI:** 10.1371/journal.pone.0252389

**Published:** 2021-06-10

**Authors:** Tadesse Tolossa, Bizuneh Wakuma, Dejene Seyoum Gebre, Emiru Merdassa Atomssa, Motuma Getachew, Getahun Fetensa, Diriba Ayala, Ebisa Turi

**Affiliations:** 1 Department of Public Health, Institute of Health Science, Wollega University, Nekemte, Ethiopia; 2 Department of Nursing, Institute of Health Science, Wollega University, Nekemte, Ethiopia; 3 Department of Midwifery, Institute of Health Science, Wollega University, Nekemte, Ethiopia; Azienda Ospedaliero Universitaria Careggi, ITALY

## Abstract

**Introduction:**

Despite its alarming spread throughout the world, no effective drug and vaccine is discovered for COVID-19 so far. According to WHO, the recovery time from COVID-19 was estimated to be 2 weeks for patients with mild infection, and 3 to 6 weeks for those with serious illnesses. A studies regarding the median recovery time and its predictors are limited globally and specifically in Ethiopia. Therefore, the aim of this study was to estimate the median time to recovery from COVID-19 and its predictors among COVID-19 cases admitted to WURH, Western Ethiopian.

**Methods:**

This was a hospital-based retrospective cohort study conducted among 263 adult patients admitted with COVID-19 in WURH treatment center from March 29, 2020 through September 30, 2020. Epidata version 3.2 was used for data entry, and STATA version 14 for analysis. A Cox proportional hazard regression model was fitted to determine factors associated with recovery time. A variable with P-value ≤ 0.25 at bivariable Cox regression analysis were selected for multivariable Cox proportional model. Multivariable Cox regression model with 95% CI and Adjusted Hazard Ratio (AHR) was used to identify a significant predictor of time to recovery from COVID-19 at P-value < 0.05.

**Results:**

The mean age of patient was 36.8 (SD± 10.68) years. At the end of follow up, two hundred twenty seven observations were developed an event (recovered) with median time to recovery of 18 days with IQR of 10–27 days. The overall incidence rate of recovery was of 4.38 per 100 (95% CI: 3.84, 4.99) person-days observations. Being older age (AHR = 1.59, 95% CI: 1.02, 2.49), presence of fever on admission (AHR = 1.78, 95% CI: 1.21, 2.62), and comorbidity (AHR = 0.56, 95% CI, 0.34, 0.90) were found to have statistically significant association with recovery time.

**Conclusion and recommendations:**

In general, the median recovery time of patients with COVID-19 cases was long, and factors such as older age group, presence of fever, and comorbidity was an independent predictors of delayed recovery from COVID-19. Intervention to further reduce recovery time at treatment center has to focus on patients those shows symptoms and with comorbidities.

## Introduction

Coronaviruses are a large group of viruses, some cause illness to human and some occur in animals. Rarely, animal coronaviruses can evolve and infect people and then may spread between people. Human coronaviruses cause routine seasonal respiratory virus infections. Other coronaviruses, like severe acute respiratory syndrome (SARS) and Middle East reparatory syndrome (MERS), can cause serious illness [1]. The emerging and rapidly evolving virus, the Novel Coronavirus Disease-2019 (SARS-Cov-2), was detected in Wuhan China in December 2019, and was initially related to exposure at a seafood and live animal market [2]. It spreads throughout countries and was categorized as pandemics by World Health Organization (WHO) in March 2020. Coronaviruses are respiratory viruses and most commonly spread through respiratory secretion of an infected person in close proximity [3].

The epidemiological dynamics of COVID-19 has changed dramatically over the courses of months. At the time of writing this manuscript COVID-19 has infected more than 149,359,118 people globally, and about 3,149,381 people died out, and 127,040,432 cases were recovered from COVID-19. In many African countries, the number of cases and number of death are low compared to European and American countries, this could be due to low test capacity, underreporting, and young population [4].

Knowing the COVID-19 fatality rate help us understand severity of the disease, identify risk factors and assess the quality of healthcare. There are difference in mortality rate among different groups due to age difference and comorbidity [5]. And also the COVID-19 fatality rate vary across different locations may be due to populations age structure and case-mix of infected and deceased patients [6].

The median time to recovery from COVID-19 varies among patients and settings, in which the average recovery time from COVID-19 more than 14 days for some countries and less than 14 days for others [7]. According to WHO, the recovery time is estimated to be 2 weeks for patients with mild infection and 3 to 6 weeks for those with serious illnesses [4]. On the other hand, CDC estimated people with mild to moderate spectrum of symptom and maintain home isolation have a resolution of 3 days after the fever decreased, and there was substantial improvement in respiratory symptoms, even without use of medication. A study conducted in treatment center found in Ethiopia showed that, the rate of prognosis from COVID-19 for asymptomatic cases was higher when compared to symptomatic COVID-19 cases, and this study reported the average recovery time of 16 days [8]. Another study conducted in Kotebe treatment center of Ethiopia revealed the media time to recovery from COVID-19 was 19 days and it ranges from 2–71 days [9].

A comprehensive literature review showed that age greater than 65 years, being hypertensive (up to 40% of patients), diabetics, obesity, cardiovascular and lung disease are possible risk factors for delayed recovery from COVID-19 [10]. In addition, presence of clinical manifestation on admission also associated with delayed recovery from COVID-19 [8].

Some studies showed improved survival of covid-19 patients depends on quality of health care services like, patient management, timing of admission, understanding of disease progress and expand use of steroids [11]. Though some scientific researches have been published, many aspects of covid-19 still need more detailed valid and reliable information. This retrospective cohort study aimed at determining time to recovery of Covid-19 infected people and its predictors among Covid-19 patients admitted to treatment center of Wollega University Referral Hospital, Western Ethiopia.

## Methods

### Study area and study period

The study was conducted at wollega University referral hospital COVID-19 treatment center which is found in Western Ethiopia. The study period was from March 29, 2020 (the first day when the first case was admitted to this treatment center) to September 30, 2020. The last date of study period (September 30, 2020) was used as an end date because this date was the last date when only severe cases of COVID-19 cases was started admitted treatment center. The data were retrieved between October 30, 2020 and November 15, 2020.

#### Study design

An institution based retrospective cohort study design was employed.

#### Study populations

Patients who were tested positive for COVID-19 by using rRT-PCR test and admitted to WURH treatment center from March 29, 2020 to September 30, 2020 with a definite outcome (event or censored) and whose chart is available during the data collection period. Patients with incomplete outcome variable and important baseline information such as date admission and outcome occurred were excluded from the analysis.

### Sample size and sampling techniques

All Covid-19 patients admitted to the treatment centers during study period (March 29, 2020 to September 30, 2020) and fulfill inclusion criteria was included in this study. A total of 298 COVID-19 cases were admitted to WURH treatment center.

### Study variables

#### Dependent variable

The dependent variable of this study was time to recovery from COVID-19. The time was estimated in days and it is the time when the patient was diagnosed positive for COVID 19 by using rRT-PCR test to the patient was diagnosed negative for COVID 19 and discharged from hospital.

#### Independent variables

Socio demographic variables such as age, sex, marital status, residence, contact history; Diseases related variables like type of medical illness, severity, time of diagnosis, co-morbidity, types of comorbidity; Clinical and laboratory variables such as presence symptoms on admission were used as an independent variables.

#### Operational definitions

Survival time is the time in days from the patient was diagnosed positive for COVID-19 by using rRT-PCR test to the occurrence of the outcome (event/censored). Event was recovery from COVID-19 or when the patient diagnosed negative after admission to treatment center by rRT-PCR test. Censored was those patients who were not developed an event or not recovered from COVID-19 (death, referred to other HI, on treatment when the study was completed). Death is death of patients from COVID-19 while they were in the treatment center, and death recorded on card was confirmed by physician. Comorbidity (Yes/No) was co-existence of one or more diseases with Covid-19 cases “Yes” and, if not it was considered as “No”.

### Data collection tools and procedure

Data were collected from registration logbook, COVID-19 intake forms and medical cards of patients. The data extraction tool was prepared from COVID-19 patient medical cards and log-book that is currently used by the COVID-19 treatment center of the hospital. The checklist consists of socio demographic related variables, diseases and past medical related variables, clinical and laboratory variables. Trained health professionals who have been working in the treatment center was extracted the data. During data collection time, the outcome was confirmed by reviewing the chart which was recorded by physician.

### Data management and analysis

Epidata version 3.2 was used for data entry, and then the data was exported to STATA version 14 for further analysis. Before analysis, data was cleaned, edited by using simple frequencies and cross tabulation; re-categorization of categorical variables and categorization of continuous variables was done to be suitable for analysis. Descriptive non-parametric survival analysis such as Kaplan Meier survival curve was used for the estimation survival probability. Days were used as time scale to calculate median time to recovery. Log rank test was used to test any difference in survival probability in categorical covariates.

A cox proportional hazards regression model was used to determine factors associated with recovery time. Factors associated with recovery time at p-value < 0.25 in bivariable cox regression were selected for multivariable cox regression analysis. Adjusted Hazard Ratios (AHR) with 95% confidence intervals was computed and statistical significance was declared when it is significant at 5% level (p value < 0.05). To assess model adequacy for proportional hazard model, proportional hazard assumption was checked by log-log plot and global test, and overall model adequacy of proportional hazard model was assessed by using cox snell residual graph.

### Ethical consideration

Ethical clearance was obtained from Wollega University research review board. Formal letter of cooperation was written to WURH treatment center and permission was obtained from the hospital administration. Personal identifiers were not used on data collection checklist.

## Results

### Description of study participants

From March 29/ 2020 through September 30/ 2020, a total of 298 patients with COVID-19 were admitted to Wollega University referral hospital treatment center. Of the total patient cards, 35 patient cards were excluded from analysis due to unregistered outcome (event, censored, date of admission, date discharge and other baseline data incomplete). Finally, 263 patient cards with complete data were included in final analysis.

### Socio-demographic characteristics of patients

The mean age of patient was 36.8 (SD± 10.68) years. The majority (46.4%) of patient’s age were ≤25 years. More than half, (57%) of patients were resides in Nekemte city, and around 62% of patients were male. More than one third of patients (33.8%) had no contact history and 22.4% of patients had known contact history (Table 1).

**Table 1:** Socio-demographic characteristics of COVID-19 cases admitted to Wollega University Referral Hospital, 2020.

Variables	Category	Survival status		Total
		Recovered	Censored	
		No	No	No (%)
**Age**	≤25	103	19	122 (46.4)
	25–40	96	13	109 (41.4)
	≥41	28	4	32 (12.2)
**Residence**	Out of Nekemte	87	26	113 (43.0)
	Nekemte	140	10	150 (57.0)
**Sex**	Male	155	7	162 (61.6)
	Female	72	29	101 (38.4)
**Contact history**	Yes	55	4	59 (22.4)
	No	84	5	89 (33.8)
	Unknown	88	27	115 (43.7)

### Baseline clinical characteristic of patients

Of the total participants, 181 (68.8%) of cases had used oxygen supplementation. Two hundred (76%) of participants had showed high fever on admission to the treatment center. One hundred 178 (77.7%) and one hundred seventy eight (67.7%) of cases had showed dry cough and throat pain on admission to treatment, respectively. More than half (53.3) had not clinically presented with severe headache on entry to hospital (Table 2).

**Table 2:** Baseline clinical features of COVID-19 cases admitted to Wollega University Referral Hospital, 2020.

Variables	Category	Survival status		Total
		Recovered	Censored	
		No	No	No (%)
**Oxygen supplemented**	Yes	56	26	82 (31.2)
	No	171	10	181 (68.8)
**High fever**	Yes	176	33	200 (76.0)
	No	66	3	63 (24.0)
**Dry cough**	Yes	151	27	178 (67.7)
	No	76	9	85 (32.3)
**Sneezing**	Yes	140	22	162 (61.6)
	No	87	14	101 (33.4)
**Throat pain**	Yes	155	23	178 (67.7)
	No	72	13	85 (32.3)
**Severe headache**	Yes	85	30	115 (43.7)
	No	142	6	148 (53.3)
**Difficulty in breathing**	Yes	63	10	73 (27.8)
	No	164	26	190 (72.2)
**Diarrhea**	Yes	42	9	43 (19.4)
	No	185	27	220 (86.6)
**Co morbidity**	Yes	21	22	49 (18.6)
	No	206	14	214 (81.4)

### Treatment outcome among patients admitted with COVID-19 in WURH treatment center

**Fig 1:**
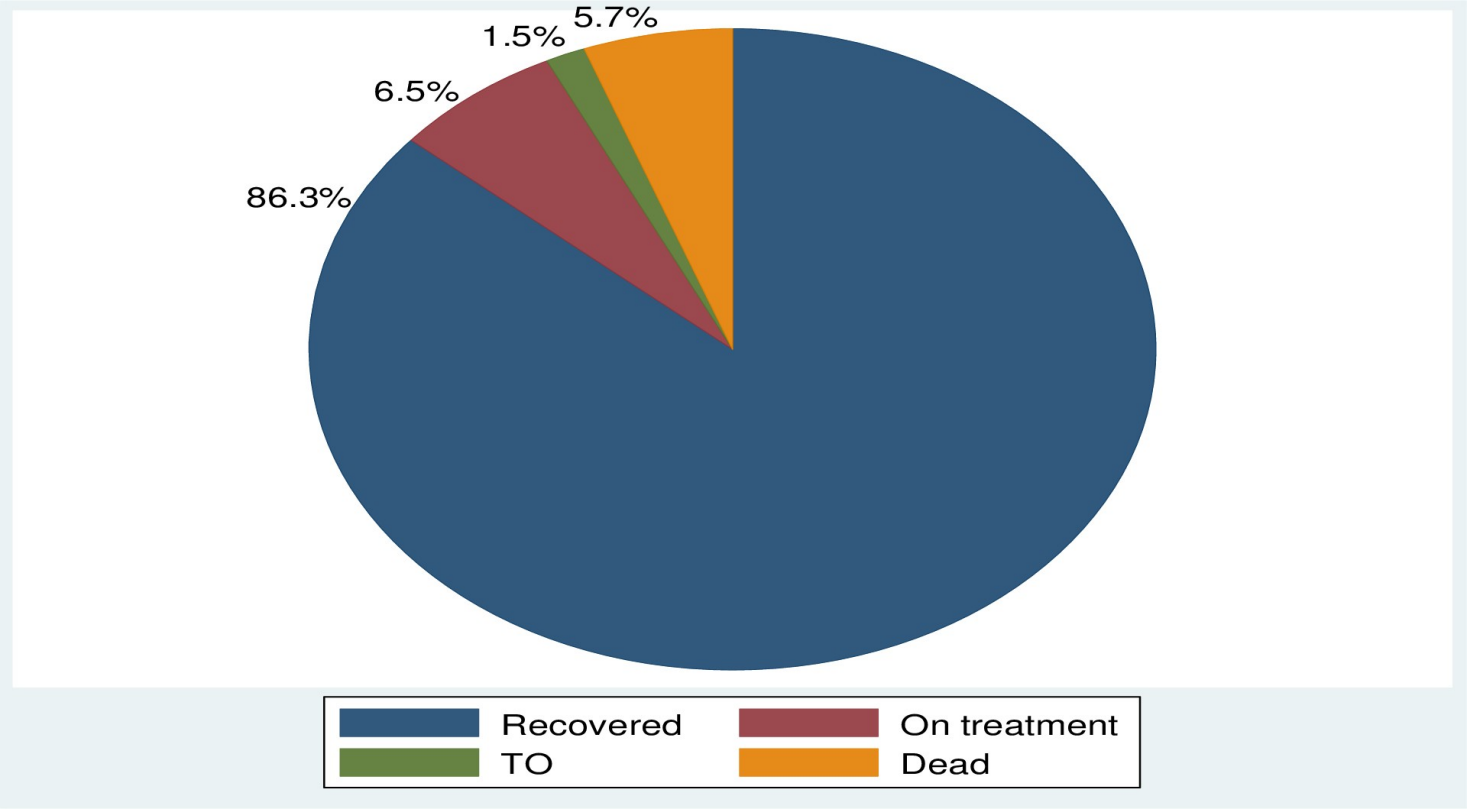
Treatment outcome among patients admitted with COVID-19 in WURH treatment.

Thirty four (12.9%) observation was censored at the end of the follow-up time. Seventeen (6.5%) were on treatment when the study was completed, 1.5% was referred for further treatment, and 5.7 were died while they were on the treatment. At the end of follow up, while 229 (87.1%) of the patients in the cohort were recovered from COVID 19 and recorded as an event (Fig 1).

### Recovery rate and median recovery time from COVID-19

A total of 263 patients were followed for a median time of 18 days. Two hundred twenty seven observations were developed an event (recovered) with median time to recovery of 18 days with IQR of 10–27 days. During follow-up time, a total of 5177 person-day risks were observed with a minimum and maximum follow-up time of 5 and 50 days, respectively. The overall incidence rate of recovery was of 4.38 per 100 (95% CI: 3.84, 4.99) person-days observations.

**Fig 2:**
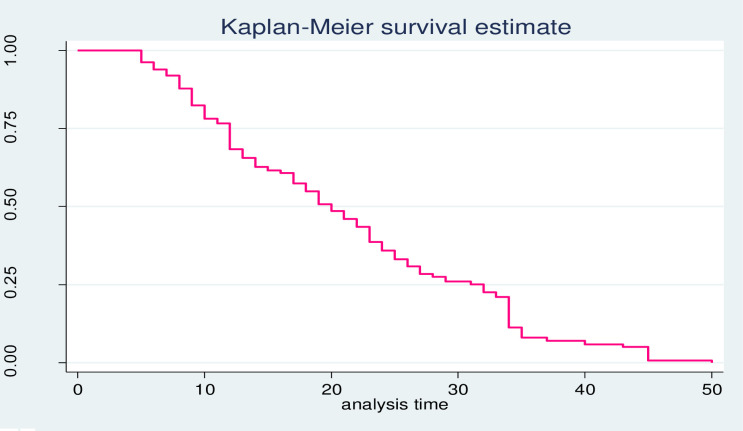
Overall Kaplan-Meier survival probability curve.

A Kaplan-Meier estimation technique was used to see the estimate of survival time. The overall graph of Kaplan-Meier survivor function depicted that the graphs decrease rapidly during the first 30 days showing most patients recovered from COVID-19 during this time (Fig 2).

**Fig 3:**
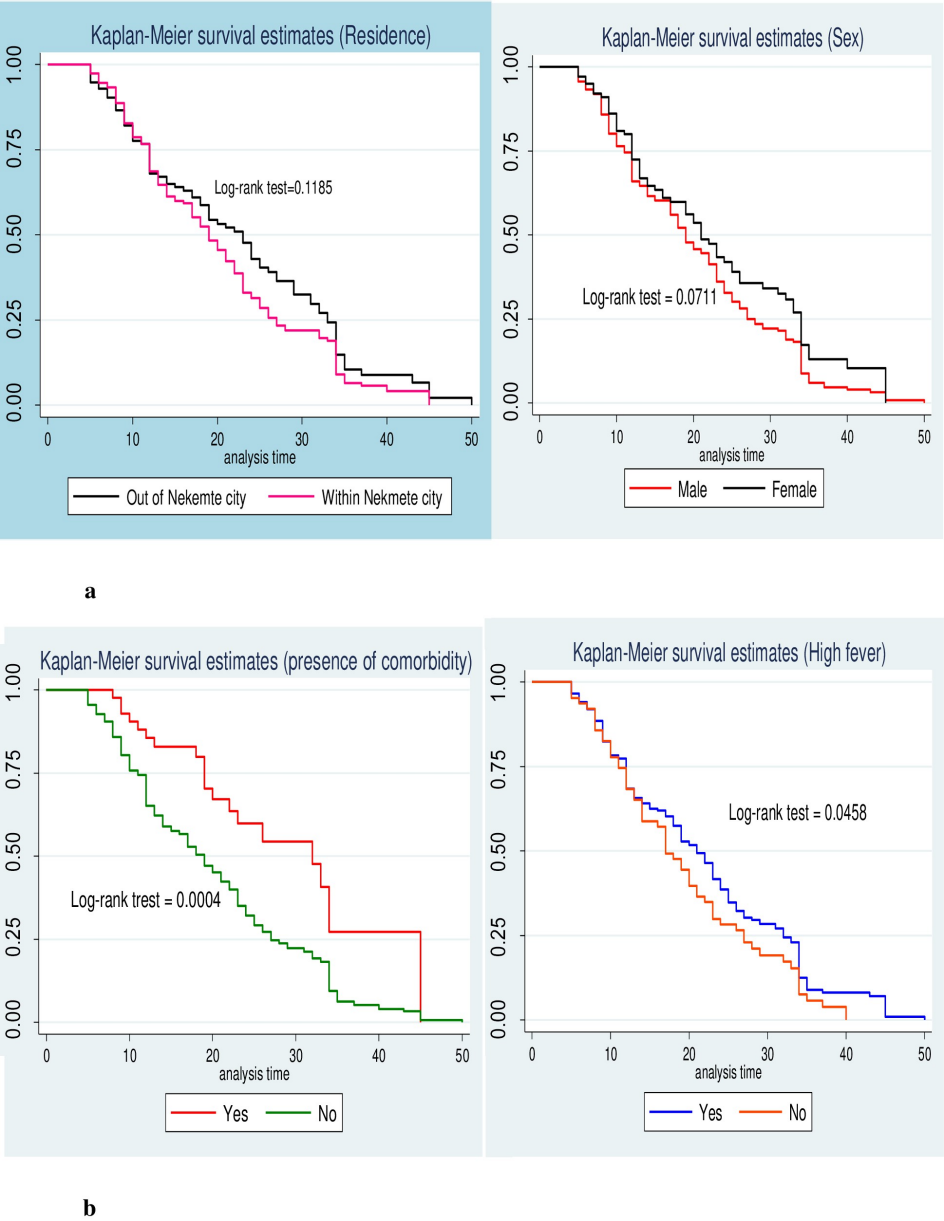
(a) Kaplan survival curve for residence and sex. (b) Kaplan survival curve for the presence fever and comorbidity.

A separate Kaplan-Meier survivor functions curve was constructed to estimates the survival time based on different covariates to see the existence of difference in recovery rate between categories of individual covariates. From Kaplan Meier survival curve of individual covariates, there were no difference in recovery rate of among male and female, and being resides in Nekemte city and out of Nekemte city (Fig 3A). However, there was a difference in survival probability/recovery rate for the covariates comorbidity and presence of fever on admission (Fig 3B). To show the significance of survival difference, log rank test was computed at 5% significance level. Accordingly, there was significance difference in survival status patients in relation to comorbidity, and presence of fever.

### Predictors of recovery time from COVID-19

Covariates that had P- value ≤ 0.25 in bivaribale cox regression analysis were selected for multivariable cox regression analysis. Residence, sex, contact history, comorbidity, presence of fever and presence of severe headache were selected for multivariable cox regression at P-value ≤ 0.25. Finally, three of the predictors (Age of patients, fever and comorbidity) were found to have statistically significant association with recovery time during multivariable cox proportional regression analysis.

Age of the patients was one of the variables that predicts recovery rate. Recovery rate of younger age groups (≤ 24) years was 1.59 times higher as compared to patients who were aged ≥41years (AHR = 1.59, 95% CI: 1.02, 2.49). Presence clinical symptom such as fever was one of the predictors that affect the recovery rate of patients from COVID-19. Patients who were not detected with fever were at higher rate of recovery than patients who were showed fever on admission (AHR = 1.78, 95% CI: 1.21, 2.62). Finally, presence of any types of comorbidity was another factor that determines the recovery time of patients with COVID-19. Presence of comorbidity lower the rate of recovery by 44% as compared to those patients who had not admitted with comorbidity (AHR = 0.56, 95% CI, 0.34, 0.90) (Table 3).

**Table 3:** Multivariable Cox regression analysis of median recovery time and its predictors among patients admitted with COVID-19 cases in WURH, 2020.

Variables	Category	Survival status		CHR	AHR	P-value
		Recovered	Not recovered			
**Residence**	Rural	87	26	1	1	
	Urban	140	10	1.22 (0.93, 1.59)	1.16 (0.88 1.53)	0.283
**Age**	≤24	103	19	1.45 (0.95, 2.22)	1.59 (1.02, 2.49)	0.039*
	25–40	96	13	1.08 (0.82, 1.43)	1.17 (0.88, 1.55)	0.276
	≥41	28	4	1	1	
**Contact history**	Yes	55	4	1	1	
	No	84	5	1.29 (0.91, 1.83)	1.10 (0.76, 1.58)	0.592
	Unknown	88	27	1.04 (0.74, 1.47)	1.07 (0.75, 1.51)	0.692
**Oxygen supplemented**	Yes	56	26	1	1	
	No	171	10	2.1 (0.72, 3.12)	1.70 (0.92, 2.23)	0.076
**Presence of fever**	Yes	176	33	1	1	
	No	66	3	1.29 (0.95, 1.73)	1.78 (1.21, 2.62)	0.003 *
**Presence of headache**	Yes	85	30	1	1	
	No	142	6	1.19 (0.90, 1.56)	1.06 (0.80, 1.40)	0.674
**Presence of comorbidity**	Yes	21	22	0.47 (0.30, 0.74)	0.56 (0.34, 0.90)	0.017*
	No	206	14	1	1	

AHR: Adjusted Hazard Ratio; CHR: Crude Hazard Ratio

*statistically significant at p<0.05.

WURH: Wollega University Referral Hospital.

### Model goodness-of-fit

**Fig 4:**
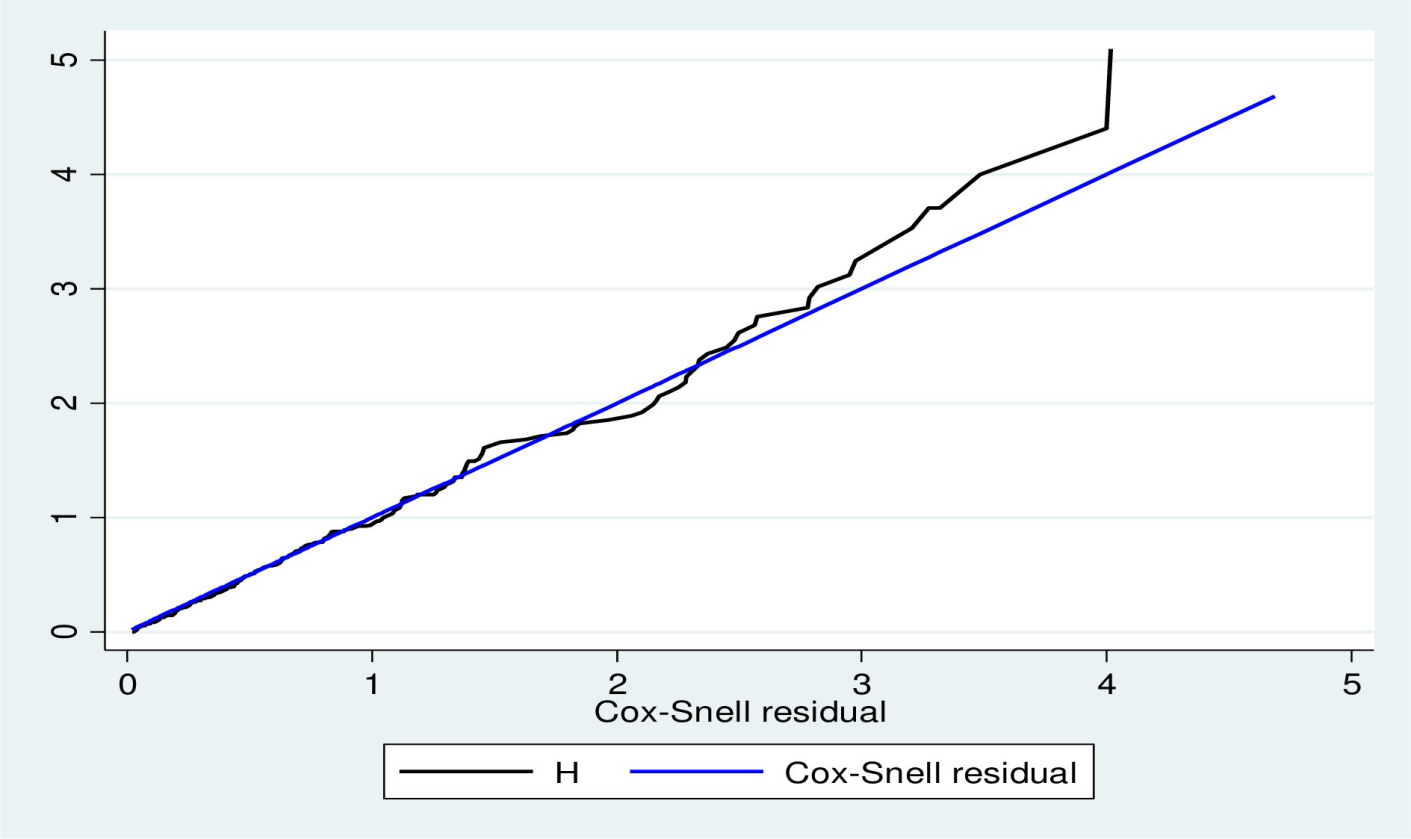
Cox Snell residual test for overall adequacy of the model.

After fitting multivariable Cox Proportional Hazard Model, adequacy of a fitted model was assessed by using cox Snell residuals. Finally, the graph of Nelson-Aalen cumulative hazard function and the cox Snell residuals variable were compared to the hazard function to the diagonal line. The hazard function follows the 45-degree line, which approximately, indicated that the model fitted the data well (Fig 4).

## Discussion

This study was aimed to determine time to recovery from novel coronavirus disease (SARS COV-2) and its predictors among patients admitted to WURH with COVID-19 cases. This study pointed out that the median time to recovery from SARS COV-2 was 18 days. This is similar to the studies done in Eka Kotebe General Hospital, Ethiopia in which viral clearance lasted for 19 days [9] and 16 days [8]. This might be due to relative similarity in care and treatment given for the patients in both study areas. Moreover, it is also is consistent with the previous study findings from Israel (20–21) days [12]. However, the median recovery time was lower in the previous studies done in Guangzhou Eighth People’s Hospital, China (12 days) [13], University of California San Diego Health (7 days) [14], Zhejiang University and the Shenzhen Third People’s Hospital, China (15 days) [15], and in Singapore (12 days) [16]. The possible reason for the observed discrepancy between the studies might be due to variation in sample size, study setting, socioeconomic characteristics, and the severity of the disease. Evidences are showing the severe the disease condition, the longer the duration of viral RNA clearance [17].

The present study found older age as independent predictor of delayed recovery time from coronavirus disease. This is consistent with previous study findings from Guangzhou, China [13], Korea [18], Wuhan Pulmonary Hospital, China [19], Shenzhen, China [15], three hospitals in Wuhan, China [17], and Qingdao, China [20]. This might be attributed to older age-related severity progression of COVID-19 cases which in turn leads to either death or delayed duration of viral clearance in elderly patients [21]. Moreover, it might be due to the fact that older age is not without comorbid conditions which are among the major risk factor of lower recovery rate form coronavirus disease and even death related to COVID-19. Besides, older age is associated with degeneration of pulmonary function and compromised immunity that contributes for severe COVID-19 cases and poor clinical outcomes.

The current study has also demonstrated that patients with comorbid condition had 44% lower odds of recovery rate from coronavirus disease compared to their counterparts. Similarly, existing evidences are supporting the present study finding, for instance, the study done in Italy [22], Fairfield General Hospital, Bury, UK [23], Wuhan Pulmonary Hospital, China [19], Jin Yin-tan Hospital and Tongji Hospital [24], and Wuhan, China [15] claim comorbid conditions majorly cardiovascular diseases attributed to the delayed duration of recovery from SARS COV-2 cases. Furthermore, our study has also identified absence of fever as a good prognostic factor of COVID-19 cases. This is in line with study conducted in Eka Kotebe treatment center of Ethiopia, in which presence of clinical manifestation on admission prolong the time of recovery from COVID-19 [8]. This is also supported by the study finding from Changsha, China [25]. This could be due to the fact that the function of respiratory system is dependent on body temperature variations [26]. This can be explained that an increment in body temperature results in increment in respiratory rate which increases the pulmonary work load eventually leading pulmonary insufficiency and lower recovery rate [26].

### Limitations

The study employed advanced statistical model for analysis. However, as the unregistered outcome and incomplete baseline data were excluded from the analysis, the reviewed records might lack very important variables that could influence recovery rate from coronavirus disease. Besides, the subjects made self-report of previously diagnosed medical illness which was considered as comorbidity.

## Conclusion

In general, this study found the prolonged recovery time from coronavirus disease. The study revealed that older age, fever at admission, and having at least one comorbid condition as a poor prognostic factors of novel coronavirus disease. Thus, elders and individuals with comorbidity has to get due attention to prevent infection by the virus. Moreover, elders and patients with comorbidity should get priority in management of coronavirus disease in order to enhance good clinical outcome.

## Supporting information

S1 File Dataset. (DTA)
